# Research on Field Weed Target Detection Algorithm Based on Deep Learning

**DOI:** 10.3390/s26020677

**Published:** 2026-01-20

**Authors:** Ziyang Chen, Le Wu, Zhenhong Jia, Jiajia Wang, Gang Zhou, Zhensen Zhang

**Affiliations:** 1 Xinjiang Space-Air-Ground Integrated Intelligent Computing Technology Laboratory, Changji 831100, China; 2 School of Computer Science and Technology, Xinjiang University, Urumqi 830049, China; 3 Xinjiang Key Laboratory of Signal Detection and Processing, Urumqi 830046, China

**Keywords:** deep learning, object detection, YOLO, weeds, occlusion, overlap

## Abstract

Weed detection algorithms based on deep learning are considered crucial for smart agriculture, with the YOLO series algorithms being widely adopted due to their efficiency. However, existing YOLO algorithms struggle to maintain high accuracy, while low parameter requirements and computational efficiency are achieved when weeds with occlusion or overlap are detected. To address this challenge, a target detection algorithm called SSS-YOLO based on YOLOv9t is proposed in this paper. First, the SCB (Spatial Channel Conv Block) module is introduced, in which large kernel convolution is employed to capture long-range dependencies, occluded weed regions are bypassed by being associated with unobstructed areas, and features of unobstructed regions are enhanced through inter-channel relationships. Second, the SPPF EGAS (Spatial Pyramid Pooling Fast Edge Gaussian Aggregation Super) module is proposed, where multi-scale max pooling is utilized to extract hierarchical contextual features, large receptive fields are leveraged to acquire background information around occluded objects, and features of weed regions obscured by crops are inferred. Finally, the EMSN (Efficient Multi-Scale Spatial-Feedforward Network) module is developed, through which semantic information of occluded regions is reconstructed by contextual reasoning and background vegetation interference is effectively suppressed while visible regional details are preserved. To validate the performance of this method, experiments are conducted on both our self-built dataset and the publicly available Cotton WeedDet12 dataset. The results demonstrate that compared to existing algorithms, significant performance improvements are achieved by the proposed method.

## 1. Introduction

Weed detection is regarded as one of the key tasks in smart agriculture, as it exerts a significant impact on crop yield and quality. In recent years, significant progress has been made in weed detection technology based on deep learning, but maintaining high detection accuracy while being lightweight remains a major challenge. Currently, deep learning algorithm models are being improved by numerous scholars, and attempts are being made to apply them to weed detection tasks. Among them, significant progress has been made by YOLOv5 in object detection, which is known for its ease of use, robustness, and flexibility, with several key innovations being introduced that have led to its widespread adoption in edge deployment scenarios [1]. The C2f structure was introduced by YOLOv8 to enhance accuracy, flexibility, and efficiency based on the success of previous versions [2]. A hybrid small-object detection method, SCO-YOLOv8s, was proposed by Zhao et al., integrating Swin Transformer and CBAM attention mechanisms into the YOLOv8 framework to enhance global and local feature representation [3]. YOLOv9 was proposed by Wang et al., and PGI and GELAN structures were designed, demonstrating the advantages of being lightweight, fast, and accurate [4]. It was found by Zhao et al. that DETR resonates with the fundamental principles of Faster R-CNN and RPN-REFINER design, thus benefiting from end-to-end detection [5]. An integral pre-training framework based on masked image modeling (MIM) was proposed by Tian et al. on the basis of Mask R-CNN, which provides multi-stage supervision for feature pyramids [6]. VMF-SSD (V-space based multi-scale feature fusion SSD) was proposed by Tian et al., which aims to extract more reliable multi-scale feature representations [7]. A hierarchical dense active supervision method, RT-Dtrv3, was proposed by Wang et al. based on RT-Detr to overcome the problem of insufficient decoder training [8]. The anchor attention mechanism was combined with YOLOv11 by Li et al., and the LIDAR module was integrated for precise 3D reconstruction of scenes, effectively enhancing feature extraction capability [9]. A novel effective trajectory prediction framework, Mamba (Tamba), was proposed by Huang et al. based on the selective state space (SSM) model to address the potential decrease in prediction accuracy from modification of the attention mechanism. The self-triggering mechanism in the encoder architecture was redesigned to achieve linear time complexity [10]. The mathematical consistency between Mamba’s internal linear attention and SSM was utilized by Gu et al. to propose a novel model based on the newly designed ACL structure, which preserves the global perspective while improving computational efficiency. A simple and powerful local enhancement module with multi-scale dilated convolutions was also designed to extract rough and fine features and improve the recovery of local details [11]. An effective convolution module, SFS-CONV, was proposed by Li et al. for SAR object detection, and a lightweight SAR object detection network, SFS-CNET, was constructed to reduce computational resources [12]. An attention-centered YOLO framework, namely YOLOv12, was proposed by Tian et al., which matches the speed of previous CNN-based frameworks while utilizing the performance advantages of attention mechanisms. All popular real-time object detectors are accurately outperformed by YOLOv12 with competitive speed [13]. A novel multifunctional fusion (PTMF) network based on point transformers was proposed by Pan et al., which enhances the global contextual feature extraction capability [14]. Although improved accuracy has been achieved by existing methods, their generalization ability in complex real-world scenarios still needs to be strengthened. In fact, factors such as different growth stages, temperature, light, nutrients, and water can alter the plant growth environment in different regions, leading to differences in weed morphology. Differences among different individuals of the same weed are caused by these factors, making subtle changes difficult to detect. Additionally, some weed species already have high similarity, such as quinoa and gray-green quinoa, and coupled with individual morphological differences, the difficulty of detection is further increased. Furthermore, although significant progress has been made by the algorithm models in the above literature in detecting individual weeds, high accuracy is still difficult to maintain while being lightweight in complex backgrounds with occlusion and overlap. In response to the above issues, the SSS-YOLO model is proposed in this paper based on YOLOv9t, combined with the ideas of Flat U-Net, LEG Net, and SEM Net frameworks [15,16,17], to solve the problem of weed occlusion in complex environmental backgrounds. The core idea of this model is to improve detection accuracy through optimization algorithms while minimizing parameter count and computational resources to better adapt to practical scene requirements. The main contributions of this paper can be summarized as follows: (1) An SCB module is designed that uses large kernel convolution to capture long-range dependencies. It bypasses local weed areas obstructed by crops to associate with unobstructed areas and enhances the features of unobstructed areas using inter-channel relationships. (2) The SPPF module and EGAS module are integrated, with multi-scale max pooling being used to extract hierarchical contextual features, a large receptive field being utilized to obtain background information around occluded objects, and the features of local areas of weeds obscured by crops being inferred. (3) An EMSN module is proposed to reconstruct the semantic information of occluded areas through contextual inference, effectively suppressing the interference of background vegetation while maintaining the integrity of visible area details.

## 2. Related Work

Today’s object detection is mainly divided into two-stage detectors and single-stage detectors—the former follow a coarse-to-fine process, while the latter adopt a one-step detection framework. Higher recognition accuracy is achieved by the two-stage object detection model, but its recognition speed is slightly lower than that of the single-stage model. In the field of weed detection, high-speed processing is required to match recognition behaviors, and real-time performance is often regarded as an important reference indicator. Therefore, single-stage models are often adopted by scholars. Although some progress has been made in research on weed detection based on single-stage detector models, the challenge of balancing lightweight and high accuracy in real complex environmental scenarios is still faced. In field scenes with occlusion and overlap, efforts have been devoted by many scholars to optimizing the model network structure. Currently, three main improvement methods are employed, namely Simplified Attention Conv Block, Spatial Pyramid Pooling, and Feedforward Net.

### 2.1. Simplified Attention Convolution

The improvement of convolutional layers is currently an active research area pursued by many scholars. For example, Liu et al. [18] proposed a novel keyword spotting method that integrates a dynamic convolution model with a multi-feature mutual learning strategy, enabling the adaptive capture of diverse and time-varying acoustic patterns. Wang et al. [19] introduced ARConv, which employs two independent sub-networks to predict the height and width of sampling positions in an image. By dynamically learning the height and width of the convolution kernel, the shape of the convolution window can be flexibly adjusted to accommodate multi-scale targets. Chen et al. [20] developed FDConv, which utilizes the inverse discrete Fourier transform (iDFT) to convert frequency-domain weights into spatial-domain convolution kernels. This approach generates more convolution kernels with distinct frequency characteristics under a fixed parameter budget, thereby enhancing the identification and separation of features from different regions. Ma et al. [21] proposed CKGConv, which uses graph positional encoding (PE) to define the pseudo-coordinates of a graph. This allows graph convolution to be defined on a continuous pseudo-coordinate system, where the convolution kernel is treated as a continuous function mapping relative positions to kernel weights, thereby flexibly enhancing feature representation capabilities. In 2024, wavelet transform-based convolution (WTConv) was introduced, which decomposes the input into different frequency components via wavelet transform. Small-kernel convolutions are then applied separately to each component, with each convolution focusing on a specific frequency range to improve the model’s shape recognition ability [22]. Qi et al. [23] designed DSConv, inspired by the characteristics of snakes, to dynamically adapt the convolutional kernel to target structures. This method addresses the segmentation challenges of slender structures and enhances detection capability for weeds with slender morphologies, such as dog fennel and field bindweed. Zhu et al. [15] developed SCAConv, which adopts a flattened feature extraction structure to reduce information loss and improve detection performance for small and occluded targets. While such methods can improve weed detection accuracy to some extent in single-category tasks involving occlusion, they often fail to enhance accuracy based on the distinct characteristics of different weed species in multi-category scenarios.

### 2.2. Spatial Pyramid Pooling

Pyramid pooling technology is widely used as an effective method for extracting multi-scale contextual information to improve the detection of occluded areas. For example, YOLOv6 was proposed by Li et al. (2022), in which simSPPF is used with simplified convolution and ReLU activation functions to improve the computational speed of pooling [24]. YOLOv7 was proposed by Wang et al. (2022), where SPPCSPC is first convolved by concatenating different convolution kernels and stride sizes, followed by weighted fusion being performed, ultimately improving detection performance but increasing computational complexity [25]. YOLOv9 was proposed by Wang et al. (2024), in which SPPELAN uses an SP structure similar to max pooling, reducing computational and parameter complexity [4]. The grouped SPPFCSPC module was proposed by Di et al. (2023), where the idea of SPPF is borrowed and a series branch is added to three parallel max pooling layer structures to improve detection accuracy [6]. A multi-scale residual spatial pyramid pool module based on the attention mechanism (RESASPPP) was proposed by Feng et al. (2025), where the attention mechanism is integrated with ASPP to effectively capture detailed information across different scales [26]. ESPPNet was proposed by [27], in which PSPP is divided into two branches. More nonlinear expressions of input features are gradually obtained through a series of convolutional layers in one branch, while rich multi-scale features are generated through max pooling and group max pooling in the other branch to improve detection accuracy. A Cross Channel Pyramid Pooling (CCPP) was designed by [28] to aggregate multi-scale pixel contextual information. The following two significant advantages in performance are presented by this type of method: detection accuracy is significantly improved by some methods, while model lightweighting is successfully achieved by others, but high accuracy and low computational resources still cannot be balanced.

### 2.3. Feedforward Network

The improvement of feedforward networks has also been employed by many scholars as a method to solve occlusion problems. For instance, Light3R-SFM was proposed by [29], where traditional global optimization is replaced with a learnable attention mechanism to effectively capture multi-view constraints across images. A sparse scene map is constructed using the shortest path tree guided by retrieval scores, thereby reducing computational resources. FRESA was developed by [30], where diffusion models are utilized to improve generation quality, combined with non-rigid alignment techniques to reduce errors. Flexible configuration of error correction steps is supported, and cross-scene generalization ability is enhanced. OmniSplat was introduced by [31] for fast feed 3D Gaussian generation from several omnidirectional images. Flare was proposed by [32], where 3D geometric shapes are inferred from uncalibrated sparse views. A high-order network (CHNET) was developed by [33] for reducing HSI data redundancy and reconstructing LiDAR features in the frequency domain to ensure the effectiveness of subsequent feature fusion. A gating mechanism is introduced to enhance the distinguishability of fused features, while their dimensionality is reduced to capture more effective deep features. SEM-NET was proposed by [17] based on the Visual State Space Model (SSM) visual network, where pixel-level damaged images are modeled while long-range dependencies (LRDs) are captured in the state space, thereby reducing linear computational complexity. Although representation ability is enhanced through nonlinear processing by these methods, problems still exist with slow weight updates in deep networks and unstable training caused by excessive weight fluctuations. In this paper, the proposed method differs from previous approaches in the following aspects:

(1) Performance optimization is achieved in existing simplified attention convolution methods by embedding attention modules in standard convolution structures, characterized by single dimensionality and a single residual connection method. However, a dual attention mechanism is employed for joint optimization in this paper, where two residual modes are utilized at two output terminals, enabling flexible adaptation to different detection tasks.

(2) The edge Gaussian aggregation method is combined with multi-scale pooling for the first time in this paper. Unlike approaches where inputs of different sizes are processed through multi-scale pooling strategies, more focus is placed on the edge features of leaves, and better association is achieved for features of weed areas obscured by crops.

(3) A context consistency strategy is constructed in this paper, and dynamic gating is designed through occlusion masks, where weights can be automatically adjusted, unlike static gating mechanisms requiring manual weight adjustment. A dual-path structure is also designed to address the scale limitations inherent in a single-path approach.

## 3. Method

### 3.1. Overall Framework

**Figure 1:**
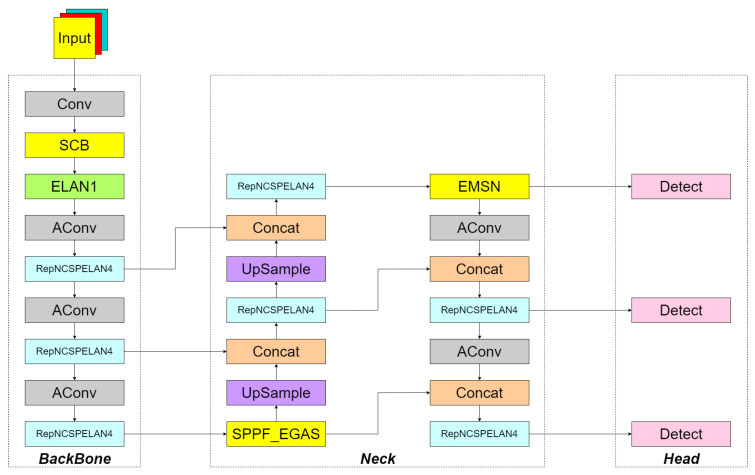
SSS-YOLO overall framework.

To address the aforementioned issues, the SSS-YOLO model is proposed in this paper, which is based on YOLOv9t and incorporates the ideas of the following three frameworks: Flat U-Net, LEG-Net, and SEM-Net. The overall structural diagram is presented in Figure 1. YOLOv9t, a model introduced in 2023 within the YOLO series, is characterized by advantages such as a small parameter count, low computational demand, and fast detection speed, demonstrating exceptional performance in target detection tasks with simple backgrounds. However, in complex environmental backgrounds, unsatisfactory results are caused by issues such as occlusion and weed overlap. To balance speed and accuracy, a shallower Backbone is adopted by YOLOv9t, which results in insufficient extraction of local features for occluded targets. When only the edge of a leaf is visible, high-level semantic features cannot be activated. Additionally, weak multi-scale feature fusion capability leads to a surge in missed detection rates when small targets are occluded. The number of channels is reduced and the attention mechanism is simplified by YOLOv9t, making it unable to dynamically suppress interference features in occluded areas. Insufficient differentiation is also shown for overlapping targets, resulting in an increased false detection rate when crops and weeds have similar colors. Therefore, improvements and optimizations are made in the backbone and neck regions in this paper to address occlusion and overlap issues. An SCB module is designed to capture long-distance dependencies using large kernel convolutions, where local areas of weeds occluded by crops are bypassed and associated with unoccluded areas, while features of unoccluded areas are enhanced using inter-channel relationships. The SPPF and EGAS modules are fused into an SPPF_EGAS module, through which hierarchical contextual features are extracted using multi-scale max pooling and background information around occluded objects is obtained by utilizing a large receptive field, enabling inference of features in local areas of weeds occluded by crops. An EMSN module is proposed to reconstruct semantic information of occluded areas through contextual reasoning, where interference from background vegetation is effectively suppressed while the integrity of details in visible areas is maintained.

The SCB is responsible for long-range dependency modeling. Its output feature maps are enhanced by SPPF_EGAS for multi-scale edge enhancement and then processed by EMSN for contextual reconstruction and occlusion reasoning, forming an end-to-end feature enhancement pathway.

### 3.2. Spatial Channel Conv Block Module

**Figure 2:**
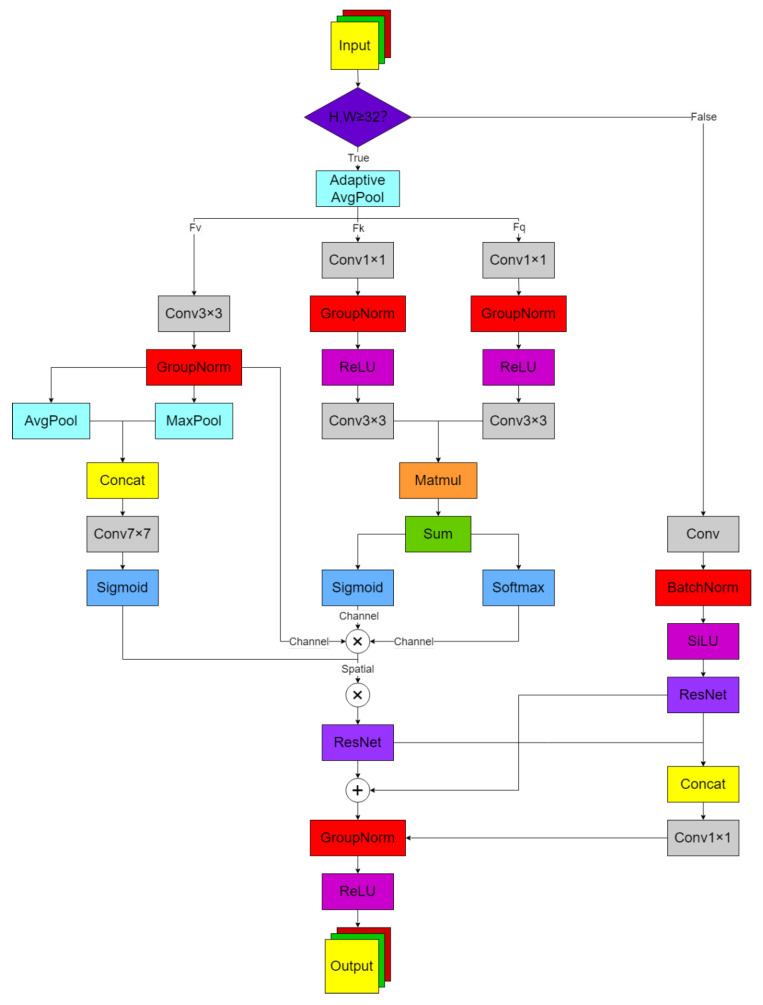
Spatial Channel Conv Block module.

In response to the weak ability of YOLOv9t to capture important features, the SCB module is proposed in this paper, with its network structure diagram shown in Figure 2. Noisy channels are suppressed and effective channels are enhanced through channel attention $\left(\alpha_{\mathrm{Softmax}}+\beta_{\mathrm{Sigmoid}}\right)$, enabling feature filtering in occluded scenes. Long-range dependencies are captured by a 7 × 7 spatial attention kernel, with key regions being highlighted to achieve small target image restoration. Computational efficiency at different resolutions is balanced by automatic switching between full attention and lightweight convolution paths, achieving multi-scale adaptation. Adaptive fusion of soft and hard attention is enabled by learnable alpha and beta parameters, allowing dynamic balance in complex environments. As shown in Figure 2, when the input feature map enters the SCB module, size judgment is first performed: when the height H and width W of the feature map are greater than or equal to the preset threshold min_Size (default 32), the complete attention path is entered; when the feature map size is smaller than min_2, lightweight convolution paths are enabled. In the complete attention path, three feature extractions are performed. For the Query branch: Grouping 1 × 1 convolution (channel compression) is applied, followed by GroupNorm and ReLU, then Grouping 3 × 3 convolution (spatial feature extraction), with Q features being output. For the Key branch: A structure symmetric to the Query branch is used but with independent parameters, with K features being output. For the Value branch: Group 3 × 3 convolution with GroupNorm is directly used, with V features being output. In lightweight convolutional paths (when the feature map is small), a single 3 × 3 grouped convolution with GroupNorm and ReLU is directly used, with the same residual connection options as the main path being maintained. Residual connection processing is then performed. Add mode: The attention output/lightweight convolution output is added element-wise with the input features. Concat mode: The output and input are concatenated along the channel dimension, with the dimension being reduced to the original number of channels through 1 × 1 convolution. Channel attention calculation is then performed: the similarity matrix is obtained by multiplying the Q and K transpose matrices, then divided by the scaling factor. Channel statistics: The similarity matrix is summed along the spatial dimension to obtain the channel description vector. Double weight generation is performed. Softmax branch: Channel competitive weights are generated (highlighting the most significant features). Sigmoid branch: Channel gating weights are generated (preserving the possibility of multiple features). Dynamic fusion: Two weights are balanced by weighting learnable parameters α and β. This process can be expressed mathematically as follows:

(1) $$\begin{array}{cc} A_{\mathrm{ch}} & =\mathrm{Softmax}\;\left(QK^{\;⊤}\right) \end{array}$$

(2) $$\begin{array}{cc} G_{\mathrm{ch}} & =\sigma \;\left(QK^{\;⊤}\right)\;\left(\mathrm{sigmoid}\;\mathrm{gate}\right) \end{array}$$

(3) $$\begin{array}{cc} W_{\mathrm{ch}} & =\frac{\alpha \;A_{\mathrm{ch}}+\beta \;G_{\mathrm{ch}}}{\alpha +\beta +\epsilon }\;(\mathrm{dynamic}\;\mathrm{balance}\;\mathrm{weight}) \end{array}$$

Spatial Attention Calculation is conducted. Dual pooling: Channel average pooling and max pooling are performed on the V features separately, resulting in two 1 × H × W feature maps. Feature concatenation: The dual pooling results are concatenated along the channel dimension. Spatial weight generation: A 7 × 7 convolution followed by Sigmoid activation is applied to generate spatial attention maps. Dual Attention Fusion: The channel attention weights are multiplied by the spatial attention weights pointwise, and the resulting weights are applied to the V features through weighted multiplication. This is followed by Output Processing. Normalization: GroupNorm processing is performed on the added or concatenated features. Activation: Non-linear activation is achieved through ReLU. Output generation: The final enhanced feature map is produced, maintaining the same dimensions (C × H × W) as the input.

### 3.3. Spatial Pyramid Pooling Fast_Edge Gaussian Aggregation Super Module

**Figure 3:**
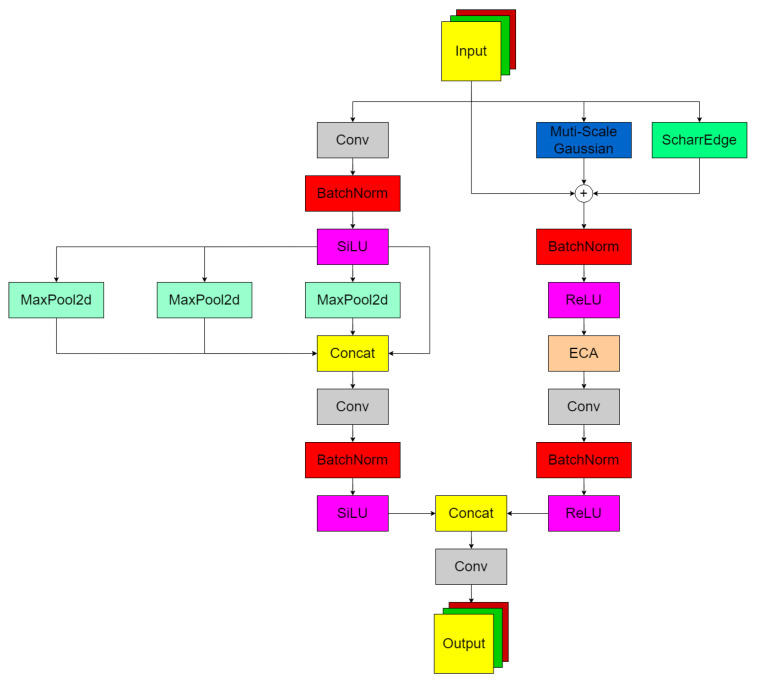
Spatial Pyramid Pooling Fast_Edge Gaussian Aggregation Super module.

The SPPF_EGAS module (see Figure 3) is constructed as a composite neural network architecture where multi-scale pyramid pooling (SPPF) and edge-guided attention mechanisms (EGAS) are integrated in a collaborative manner to address the degradation of feature representation caused by scale variations and local occlusions in complex visual environments. Multi-scale context fusion is achieved through hierarchical receptive fields that are built using Serial Repetitive Maximum Pooling, enabling the capture of multi-level feature responses spanning from local details to global semantics while effectively mitigating feature matching bias resulting from target scale diversity. Contextual information at different scales is explicitly preserved through implementation of a hole pooling strategy (kernel = k, stride = 1, padding = k/2), with the spatial resolution of feature maps being consistently maintained throughout the processing pipeline.

SPPF Branch:

(4) $$Y_{SPPF}={\mathrm{Conv}}_{1\times 1}\left(\mathrm{Concat}\left[Y_{0},Y_{1},Y_{2},Y_{3}\right]\right)\in R^{B\times C_{1}\times H\times W}$$

Edge-enhanced feature recalibration is achieved through the Edge Perception Branch (EGAS), where first-order gradient features are extracted by the Scharr operator, resulting in enhanced separation between low-frequency and high-frequency components at the target–background boundary while texture noise interference in occluded areas is effectively suppressed. Multi-scale Gaussian smoothing and edge detection are mutually complemented, with detail preservation and noise robustness being balanced through carefully selected scale parameters (σ∈[0.8,1.2]). Dynamic channel attention fusion is implemented through efficient channel attention (ECA), where channel-level weights are allocated to fused features, leading to an enhanced response strength of target-related features and weakened ineffective activation in occluded regions. EGAS Branch:

(5) $$G_{\sigma }\left(X\right)=\frac{1}{2\pi \sigma^{2}}\exp\left(-\frac{x^{2}}{2\pi \sigma^{2}}\right)*X$$

(6) $$Y_{Gauss}=\frac{1}{2}\left(G_{0.8}\left(X\right)+G_{1.2}\left(X\right)\right)$$

(7) $$Y_{Edge}=\sqrt{E_{x}^{2}+E_{y}^{2}+\epsilon }$$

(8) $$Y_{Fuse}=\mathrm{BN}(\mathrm{ReLU}\left(X+Y_{Gauss}+Y_{Edge}\right))$$

Finally, branch feature interaction is achieved through 1 × 1 convolution, forming a more discriminative mixed representation.

(9) $$SPPF\_EGAS\left(X\right)=f_{Fusion}\left(f_{SPPF}\left(X\right),f_{EGAS}\left(X\right)\right)$$

where the input feature map is $X\in R^{BC_{1}HW}$, the Gaussian filtering scale is σ∈0.8,1.2, YGauss is the Gaussian filtering component, YEdge is the edge detection component, fSPPF is the composite function of the SPPF branch, fEGAS is the composite function of the EGAS branch, and $f_{Fusion}$ is the fusion function (1 × 1 convolution).

### 3.4. Efficient Multi-Scale Spatial-Feedforward Network Module

**Figure 4:**
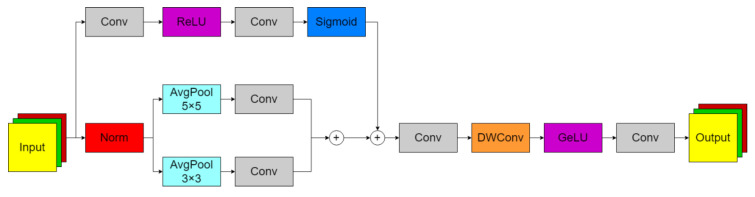
Efficient Multi-Scale Spatial-Feedforward Network module.

An explicit occlusion modeling mechanism is established (see Figure 4), where the ability to automatically generate spatial occlusion masks from feature maps is constructed through the addition of parallel occlusion perception branches. This branch is implemented with a two-level convolutional structure: the channel dimension is first compressed by 3 × 3 convolution for local feature extraction, followed by restoration of the original channel number through another 3 × 3 convolution, with spatial attention maps being generated via Sigmoid activation. The output occlusion mask serves to quantify the probability of each pixel position being obscured by vegetation, enabling explicit differentiation between occluded and visible regions. Heterogeneous multi-scale feature fusion is achieved through a dual-path structure designed to overcome the scale limitations inherent in traditional single-path approaches. In the main path, local geometric features are preserved through 3 × 3 average pooling, while morphological perception capability is constructed using two-layer 3 × 3 convolution, specifically targeting the serrated edges of weed leaves. The small-scale pathway employs global average pooling for spatial dimension compression, with long-range dependencies being established via 1 × 1 convolution, effectively modeling cross-regional occlusion contexts. Scale alignment is accomplished through channel-wise feature replication, followed by element-wise addition to form compensation features. A dynamic feature enhancement strategy is implemented through an adaptive feature fusion method based on occlusion masks. Weighted summation is performed between the original feature map and multi-scale compensation features, with weights being dynamically controlled by the occlusion mask. In identified occluded regions (mask values approaching 1), compensation features are predominantly utilized, while original features are retained in visible areas (mask values approaching 0), achieving pixel-level feature restoration.

(10) $$EMSN\left(X\right)=W_{7}*\left[\mathrm{GELU}\left(W_{6}*{\left[W_{3}*\left(X⊙(1-M)+C⊙M\right)\right]}_{c^{\prime }}\right)⊙\left[W_{6}*{\left[W_{3}*\left(X⊙(1-M)+C⊙M\right)\right]}_{c^{\prime }}\right]\right]$$

where $W_{i}$ are Grouping convolution weights, i∈{1,2,3,4,5,6,7}, ∗ is the convolution operation, and ⊙ is Element-wise multiplication.

This mechanism enables the detail integrity of visible areas to be maintained by the model, with the local semantic information of weeds obscured by crops being reconstructed through contextual inference, while the interference from background vegetation is effectively suppressed.

## 4. Experiment

### 4.1. Dataset

To validate model generalization, experiments were conducted on both the public CornWeed12 dataset and a custom-built dataset to enable comprehensive performance evaluation. The CornWeed12 dataset comprises common cotton field weeds collected across multiple southern U.S. states. Image acquisition was performed using smartphones and handheld digital cameras under natural lighting conditions between February and October 2021, ultimately establishing a large-scale weed dataset. The following twelve weed species were documented: Abutilon theophrasti, Alligatorweed, Asiatic_Smartweed, Barnyard_grass, Bidens pilosa, Billygoat_weed, Black_nightshade, Ceylon_spinach, Chinese_knotweed, Cocklebur, Common Dayflower, and Crabgrass. Given the geographical limitations of public datasets, an additional six-category weed dataset with regional characteristics from Xinjiang was developed to extend the public dataset. Data collection was carried out at Huaxing Farm (86.96° E, 44.23° N) in China’s Xinjiang Uygur Autonomous Region, covering representative cotton and corn fields. Image capture was implemented using a vivo Z5 digital camera and DJI Mavic2 drone (4096 × 3027 resolution, 4:3 aspect ratio), mounted on a mobile tripod platform to maintain controlled shooting parameters (400–600 mm distance, 45–90° angles). From 2085 screened images, the following six weed species were documented: *Amaranthus retroflexus* L., *Amaranthus albus* L., *Chenopodium album* L., *Oxybios glauca* L., *Portulaca oleracea* L., and *Setaria viridis* L. The dataset was partitioned into training and validation sets at an 8:2 ratio, with preferential allocation for minority classes to partially address sample imbalance. Unified weed annotation was performed using LabelImg with rectangular bounding boxes, initially stored in XML format and subsequently converted to TXT format for experimental compatibility.

### 4.2. Environment Setup

The experimental parameters are presented in Table 1. The configuration employed for these experiments consisted of an Nvidia RTX 3050 Ti 4 G GPU, with CUDA 11.7, Torch 1.12.0, and TorchVision 0.13.0 being utilized as the software environment. A cosine function was implemented as the learning rate scheduler, while stochastic gradient descent (SGD) was selected as the optimization algorithm, with its weight decay factor being set to 0.0005. Due to GPU memory constraints, the batch size was limited to 6, with the training epoch count being fixed at 500 cycles. All experiments were conducted at a resolution of 640 × 640 pixels. To validate model generalization, comparative testing was performed on both the custom-built dataset and the public CornWeed12 dataset, enabling comprehensive performance evaluation across different data sources.

### 4.3. Attention Comparison Method

To verify that the proposed SCB module outperforms other simplified attention–convolution modules, this section will incorporate SE, CBAM, and CA modules into the YOLOv9t architecture for comparative analysis in Table 2. As can be seen from the table, the SCB module proposed in this paper outperforms the other modules in all of the following five evaluated aspects: precision, recall, parameter count, mAP@50, and mAP@50:95.

### 4.4. Comparison Method

The comprehensive performance of the proposed SSS-YOLO model was verified through comparative experiments conducted with eleven object detection models on a custom-built dataset, including YOLOv5n, YOLOv8n, YOLOv9t, YOLOv9s, YOLOv9m, YOLOv10n, YOLOv10s, YOLOv10m, YOLOv11n, YOLOv11s, YOLOv11m and YOLOv12n.

### 4.5. Evaluation Index

Evaluation of the object detection model was performed using multiple metrics, including maximum average accuracy (mAP50 95), mean average accuracy (mAP50), precision (Precision), recall (Recall), number of operations per second (GFLOPs), and parameter count (Params).

### 4.6. Ablation Experiment

The performance of the SSS-YOLO algorithm for weed detection was evaluated through ablation experiments focusing on the following three key modules: SCB, SPPF_EGAS, and EMSN, with the experimental results presented in Table 2 demonstrating significant improvements achieved through various structural and algorithmic enhancements. In the backbone network architecture, the conventional second convolution layer was successfully replaced with the Spatial Channel Enhancement Convolution Module (SCB), resulting in substantially improved performance metrics while maintaining the original parameter count, despite a measured increase in computational complexity. Specifically, a 7.4% enhancement in precision was observed along with a 6% improvement in recall rate, while the mean average precision (mAP50%) and maximum average precision (mAP50-95%) were increased by 7.0% and 6.6%, respectively, confirming the effectiveness of the proposed modifications.

The architectural modifications were systematically implemented across the network structure, beginning with the integration of the SPPFEGAS module between the backbone and neck networks, which was designed by combining characteristics from YOLOv11 and LEG Net to replace YOLOv9t’s original SPPELAN module. This modification was observed to maintain identical parameter counts and computational complexity while achieving measurable improvements of 5.6% in precision, 1.2% in recall, 2.7% in mean average precision (mAP50%), and 2.0% in maximum average precision (mAP50-95%). Subsequently, in the neck network architecture, an Efficient Multi-scale Spatial Feedforward Network (EMSN) was incorporated at the P3 layer connection between the RepNCSELAN4 and Detect components, resulting in marginal increases in both parameters and computations that were offset by performance gains of 5.6% in precision, 1.2% in recall, 3.7% in mAP50%, and 3.1% in mAP50-95%. When implemented concurrently, the SPPFEGAS and EMSN modules were found to produce combined enhancements of 4.7% in precision, 4.3% in recall, 5.0% in mAP50%, and 3.7% in mAP50-95%, despite slight increases in architectural complexity. The comprehensive integration of all three proposed modules (SCB, SPPFEGAS, and EMSN) into the SSS-YOLO framework was demonstrated to maintain baseline parameter counts while judiciously increasing computational overhead, ultimately delivering significant performance improvements across all metrics—including 7.8% higher precision, 6.5% improved recall, 8.6% enhanced mAP50%, and 7.5% greater mAP50-95% compared to the original YOLOv9t implementation.

### 4.7. Comparative Experiment

Comparative analysis revealed significant performance advantages of the proposed SSS-YOLO model when evaluated against comparable architectures. When benchmarked against YOLOv5n, YOLOv8n, YOLOv9t, YOLOv10n, YOLOv11n, and YOLOv12n models with equivalent parameter counts, SSS-YOLO was observed to exhibit only increased floating-point operations while demonstrating substantial improvements across all other metrics. Precision was enhanced by 3.5%, 3.0%, 7.8%, 5.9%, 5.7%, and 0.3%, respectively; recall rates were elevated by 5.6%, 3.9%, 6.5%, 7.5%, 3.0%, and 5.8%; mean average precision showed gains of 7.0%, 6.0%, 8.6%, 9.3%, 6.4%, and 4.9%; and maximum average accuracy was improved by 7.3%, 6.0%, 7.5%, 7.1%, 6.2%, and 5.1% across the respective model comparisons. In terms of inference speed, SSS-YOLO achieved 297 FPS, representing a 28.8% reduction compared to the fastest YOLOv5n (417 FPS) but maintaining a comparable performance with YOLOv9t (302 FPS), slightly exceeding YOLOv10n (289 FPS) by 2.8%, while trailing YOLOv8n (366 FPS) by 18.9%, YOLOv11n (376 FPS) by 21.0%, and YOLOv12n (401 FPS) by 25.9%.

Further evaluation against YOLOv9s, YOLOv10s, and YOLOv11s models with similar computational complexity demonstrated that SSS-YOLO was marginally surpassed only by YOLOv11s in precision, while outperforming all counterparts in remaining aspects. Precision improvements of 3.0% and 2.2% were recorded against YOLOv9s and YOLOv10s, respectively, accompanied by recall rate increases of 2.9%, 7.0%, and 5.2%. Parameter counts were reduced by 69.3%, 76.2%, and 79.7%, with mean average precision elevated by 4.4%, 7.3%, and 4.1% and maximum average accuracy improved by 3.1%, 5.1%, and 3.3%. In inference speed, SSS-YOLO demonstrated a 24.3% increase over YOLOv9s (239 FPS), a 67.8% increase over YOLOv10s (177 FPS), and a 57.1% increase over YOLOv11s (189 FPS).

When compared against larger-scale YOLOv9m, YOLOv10m, and YOLOv11m architectures, SSS-YOLO was found to deliver a superior performance across most metrics despite a slightly lower precision (2.6% and 2.9% reductions versus YOLOv9m and YOLOv11m). Compared to YOLOv10m, a 0.8% precision advantage was maintained, along with recall rate improvements of 3.4%, 4.9%, and 4.8%. Significant reductions were achieved in both parameter counts (88.5%, 88.4%, and 90.5% decreases) and floating-point operations (52.4%, 55.0%, and 57.8% reductions), while mean average precision was enhanced by 2.1%, 4.8%, and 2.8% and maximum average accuracy was improved by 0.7%, 2.7%, and 1.6% respectively. SSS-YOLO demonstrated substantial inference speed advantages, achieving 62.3% higher FPS than YOLOv9m (183 FPS), 172.5% higher than YOLOv10m (109 FPS), and 185.6% higher than YOLOv11m (104 FPS), while maintaining comparable or superior accuracy metrics.

### 4.8. Generalization Experiment

The generalization capability of the SSS-YOLO model was verified through comparative evaluation between the publicly available CottonWeedDet12 dataset and the custom-built six-weeds dataset, with the results presented in Table 3. The CottonWeedDet12 dataset, comprising common cotton field weeds from southern U.S. states, was constructed through image capture using smartphones or handheld digital cameras under natural lighting conditions between February and October 2021, ultimately forming a large-scale weed dataset. As evidenced in the results, a superior overall performance was observed on the CottonWeedDet12 dataset compared to the custom dataset, attributed to its less severe occlusion scenarios. Nevertheless, higher accuracy was consistently demonstrated by the proposed SSS-YOLO model when compared to existing YOLO series algorithms across both datasets. Table 4 and Table 5 reveal that SSS-YOLO exhibited enhanced performance across multiple metrics, including mAP, GFlops, and parameter count, regardless of dataset variations. Superior detection accuracy was achieved over the original YOLOv9t model on both the custom dataset and the public CottonWeedDet12 dataset, confirming the model’s robust generalization capability across different data sources.

### 4.9. Visualization Analysis

**Figure 5:**
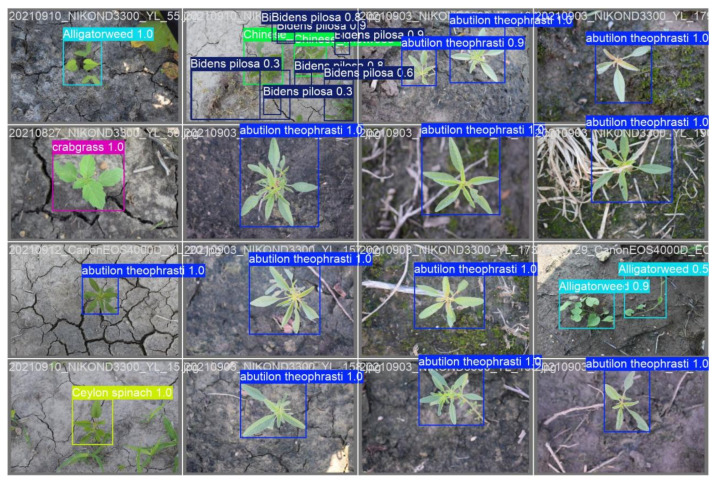
YOLOv9t.

**Figure 6:**
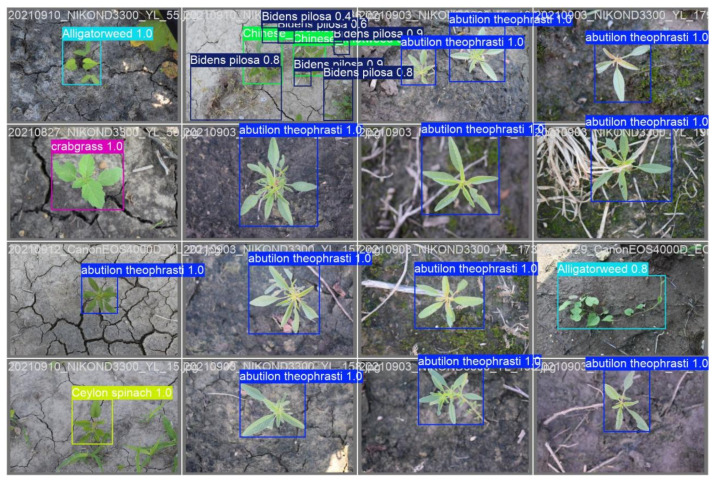
SSS-YOLO.

The optimized SSS-YOLO model demonstrates superior comprehensive recognition capabilities, delivering the most precise and thorough weed identification results in complex scenarios involving occlusion and overlapping vegetation. The comparative visualization results between YOLOv9t and SSS-YOLO on the publicly available CottonWeedDet12 dataset are presented in Figure 1. Analysis of the results reveals that while near-perfect detection accuracy (approaching 100%) is achieved by YOLOv9t (see Figure 5) for isolated weed specimens, significant performance degradation is observed when processing occluded and overlapping vegetation, with accuracy rates dropping as low as 30%. Furthermore, instances are noted where individual weeds are erroneously detected as multiple specimens, indicating that conventional lightweight YOLO algorithms remain inadequate for reliable weed detection in complex, obstructed environments. In contrast, SSS-YOLO (see Figure 6) maintains high detection precision for individual specimens while simultaneously achieving improved accuracy for challenging cases involving occluded and overlapping species such as Bidens pilosa. Notably, no instances of single-plant duplication are recorded. These findings collectively demonstrate that enhanced feature extraction capabilities are exhibited by the SSS-YOLO architecture compared to baseline implementations.

### 4.10. Experimental Results and Discussion

The analysis of all experimental results presented above reveals that existing YOLO series algorithms are characterized by high false detection rates. This limitation is attributed to the adoption of large receptive field designs in YOLO architectures, which results in insufficient capture of detailed information for small targets, particularly in dense scenarios where weed specimens overlap. Furthermore, the implementation of fixed-number bounding box predictions per grid leads to increased false detection rates when multiple or overlapping targets are present within a given area. A superior performance is demonstrated by the proposed method compared to other YOLO series algorithms, as evidenced by the experimental results. The enhanced effectiveness of this approach can be explained by the following three key factors: (1) Automatic path selection is implemented based on feature map dimensions, with dense or lightweight paths being dynamically chosen through different residual modes to accommodate varying environmental conditions. (2) Edge detail features are effectively extracted through the application of edge Gaussian aggregation methodology, enabling improved association between occluded and unobstructed regions. (3) A context consistency strategy is established, where weights are dynamically regulated through occlusion encoding to effectively suppress background vegetation interference. Consequently, successful application of this methodology is achieved across multiple weed dataset categories, demonstrating its versatility and robustness in diverse agricultural scenarios. The passive construction maintains all technical specifications while shifting focus to the methodological innovations and their demonstrated outcomes.

## 5. Conclusions

A novel object detection model named SSS-YOLO is proposed in this study for weed detection in multi-label classification domains characterized by complex environmental backgrounds with occlusion and overlap. The following three key innovations are implemented in the model architecture: (1) feature filtering and occluded image restoration are accomplished through the SCB module; (2) edge features and contextual information are effectively complemented via the SPPF_EGAS module; and (3) semantic information reconstruction of occluded regions is achieved by the EMSN model to suppress background vegetation interference. Superior detection performance is demonstrated on both the custom-built dataset and the publicly available CottonWeedDet12 dataset compared to existing YOLO series algorithms, with high detection accuracy being maintained while lightweight characteristics are preserved. The robustness and stability of the proposed methodology are comprehensively evidenced in Table 4 and Table 5. Future research efforts will be directed toward dataset category expansion to enhance generalization capabilities across different weed types.

## Figures and Tables

**Table 1:** Experimental setup.

Configuration	vivoZ5	HP Shadow Sprite 8 (PC)	Raspberry Pi 4B
CPU	Qualcomm Snapdragon 712; 6 GB + 256 GB	i5-12500H 16 G	ARM Cortex-A72 1.5 GHz (quad-core) SD Card (32 GB)
GPU	-	Nvidia RTX 3050Ti 4 G	500 MHz VideoCore VI
Camera	Posterior 48 megapixels; Resolution 4096 × 3027	-	CSI camera; 8 megapixels; Video 1080p30 resolution
Operating system	FuntouchOS 9	Windows 11	Linux 2014 64
Accelerated environment	-	CUDA 11.7	-
Library	-	Torch 1.12.0; Torchvision 0.13.0	Torch; Opencv

**Table 2:** Attention comparison experiment results.

Model	P%	R%	Params/M	Flops/G	mAP 50%	mAP50-95%
YOLOv9t	71.4	63.3	1.7	6.5	68.8	45.2
YOLOv9t + SCB	78.8	69.9	1.7	25.8	75.8	52.0
YOLOv9t + SE	73.8	65.6	3.1	12.1	70.3	47.1
YOLOv9t + CBAM	76.6	65.9	3.0	12.7	70.8	46.9
YOLOv9t + CA	77.2	67.0	3.0	12.2	71.2	48.1

**Table 3:** Generalization experiment results.

Model	P%	R%	Params/M	Flops/G	mAP50%	mAP50-95%
YOLOv5n	92.2	88.3	2.1	5.9	91.7	85.9
YOLOv8n	92.3	85.8	2.7	6.9	93.0	87.9
YOLOv9t	92.7	87.0	1.7	6.5	93.0	87.7
YOLOv9s	94.4	87.4	6.2	22.3	94.0	89.0
YOLOv10n	92.8	88.3	2.7	8.4	93.4	88.3
YOLOv10s	94.2	89.1	8.0	24.6	94.1	89.5
YOLOv11n	93.3	86.7	2.6	6.4	92.8	87.8
YOLOv11s	94.7	88.8	9.4	21.5	94.6	89.3
YOLOv12n	93.4	88.6	2.5	6.3	94.0	88.4
SSS-YOLO	95.5	89.3	1.9	28.4	95.3	91.2

**Table 4:** Ablation experiment results.

Model	P%	R%	Params/M	Flops/G	mAP 50%	mAP50-95%
YOLOv9t	71.4	63.3	1.7	6.5	68.8	45.2
YOLOv9t + SCB	78.8	69.9	1.7	25.8	75.8	52.0
YOLOv9t + SPPF_EGAS	77.0	64.5	1.7	6.6	71.5	47.2
YOLOv9t + EMSN	77.0	64.5	1.8	7.2	72.5	48.3
YOLOv9t + SPPF_EGAS + EMSN	76.1	67.6	1.9	7.2	73.8	48.9
SSS-YOLO	79.2	69.8	1.9	28.7	77.4	52.7

**Table 5:** Comparative experiment results.

Model	P%	R%	Params/M	Flops/G	FPS	mAP50%	mAP50-95%
YOLOv5n	75.7	64.2	2.1	5.9	417	70.4	45.4
YOLOv8n	76.2	65.9	2.7	6.9	366	71.4	46.7
YOLOv9t	71.4	63.3	1.7	6.5	302	68.8	45.2
YOLOv9s	76.2	66.7	6.2	22.3	239	73.0	49.6
YOLOv9m	81.8	66.4	16.6	60.4	183	75.3	52.0
YOLOv10n	73.3	62.3	2.7	8.4	289	68.1	45.6
YOLOv10s	77.0	62.8	8.0	24.6	177	70.1	47.6
YOLOv10m	78.4	64.9	16.5	63.8	109	72.6	50.0
YOLOv11n	73.5	66.8	2.6	6.3	376	71.0	46.5
YOLOv11s	81.1	64.6	9.4	21.5	189	73.3	49.4
YOLOv11m	82.1	65.0	20.1	68.1	104	74.6	51.1
YOLOv12n	78.9	64.0	2.5	6.3	401	72.5	47.6
SSS-YOLO	79.2	69.8	1.9	28.7	297	77.4	52.7

## Data Availability

Data will be made available on request.
